# Health Diagnosis of Major Transportation Infrastructures in Shanghai Metropolis Using High-Resolution Persistent Scatterer Interferometry

**DOI:** 10.3390/s17122770

**Published:** 2017-11-29

**Authors:** Xiaoqiong Qin, Tianliang Yang, Mengshi Yang, Lu Zhang, Mingsheng Liao

**Affiliations:** 1 State Key Laboratory of Information Engineering in Survey, Mapping and Remote Sensing, Wuhan University, Wuhan 430079, China; qinxiaoqiong@whu.edu.cn (X.Q.); yangms@whu.edu.cn (M.Y.); luzhang@whu.edu.cn (L.Z.); 2 Collaborative Innovation Center for Geospatial Technology, Wuhan University, Wuhan 430079, China; 3 Key Laboratory of Land Subsidence Monitoring and Prevention, Ministry of Lanf and Resources, Shanghai 200072, China; sigs_ytl@163.com; 4 Shanghai Institute of Geological Survey, Shanghai 200072, China; 5 Department of Geoscience and Remote Sensing, Delft University of Technology, 2628 CN Delft, The Netherlands

**Keywords:** large infrastructures, deformation, high-resolution InSAR, Shanghai

## Abstract

Since the Persistent Scatterer Synthetic Aperture Radar (SAR) Interferometry (PSI) technology allows the detection of ground subsidence with millimeter accuracy, it is becoming one of the most powerful and economical means for health diagnosis of major transportation infrastructures. However, structures of different types may suffer from various levels of localized subsidence due to the different structural characteristics and subsidence mechanisms. Moreover, in the complex urban scenery, some segments of these infrastructures may be sheltered by surrounding buildings in SAR images, obscuring the desirable signals. Therefore, the subsidence characteristics on different types of structures should be discussed separately and the accuracy of persistent scatterers (PSs) should be optimized. In this study, the PSI-based subsidence mapping over the entire transportation network of Shanghai (more than 10,000 km) is illustrated, achieving the city-wide monitoring specifically along the elevated roads, ground highways and underground subways. The precise geolocation and structural characteristics of infrastructures were combined to effectively guide more accurate identification and separation of PSs along the structures. The experimental results from two neighboring TerraSAR-X stacks from 2013 to 2016 were integrated by joint estimating the measurements in the overlapping area, performing large-scale subsidence mapping and were validated by leveling data, showing highly consistent in terms of subsidence velocities and time-series displacements. Spatial-temporal subsidence patterns on each type of infrastructures are strongly dependent on the operational durations and structural characteristics, as well as the variation of the foundation soil layers.

## 1. Introduction

In order to support the economic sustainable development and quality of human life, recently, Shanghai has made enormous efforts in expanding its massive infrastructure networks, especially for the roads, highways and subways. However, most of these infrastructures are, to some degree, degenerated in terms of material performance and structural functionality, resulting in huge financial and human losses. Lack of timely disaster prevention monitoring and management would accelerate the growing costs of repairs and replacements [1,2]. Moreover, since a large amount of people in Shanghai (more than 18 million) move via roads and travel by subways every day, the fast-deteriorating infrastructures may also pose significant risks to public safety. Consequently, the health diagnosis of these infrastructures is now urgently needed by civil engineers to gain up-to-date information on their structural safety, as well as to prevent and mitigate the associated risks, especially in such densely inhabited urban areas [3,4,5].

Over the past decades, Shanghai’s responsible offices spent a considerable amount of money to install different kinds of instruments to monitor the stability of terrain [3]. However, urban infrastructures are usually linear objects, with the characteristics of long distance, large-scale during the routine inspection. Therefore, it is impractical to install these expensive conventional on-structure sensors along all the infrastructures, let alone to frequently detect the overall and detailed subsidence of these linear features by those sparse survey networks. Compared with the point-measurement geodetic techniques, Interferometric Synthetic Aperture Radar (InSAR) technology furnished a non-contact tool to cost-effectively detect the millimeter level deformation at a significantly improved spatial resolution over large areas [6,7,8]. Thanks to the development of Persistent Scatterer InSAR (PSI) technique, the intrinsic issues of conversional InSAR are overcome by modeling and analyzing the persistent scatterers (PSs) with steady radar reflectivity over a long time [9,10,11,12]. Therefore, PSs are not affected by baseline decorrelation and all the acquired data can be used to generate a single master stack of interferograms even if the baselines are longer than the critical baseline [13]. Since the PSs are more abundant in urban environments, PSI is an extremely suitable method for time-series analysis of land subsidence in metropolitan areas [3,14,15,16]. The numerous archives data and intensive detectable PSs of high-resolution images (i.e., German TerraSAR-X (TSX)) make it possible to detect the existing subsidence and grasp the long-term subsidence trends of large infrastructures [17,18,19].

However, successful PSI-based studies focused more on regional subsidence monitoring in populated cities, for instance Shanghai, Tianjin and Hong Kong [20,21,22], rather than the special topic on large infrastructures subsidence mapping over an entire city. This may due to two important reasons: (1) the layover and foreshortening effects, induced by the intrinsic side-looking geometry of SAR sensors, make it difficult to distinguish the PSs that are exactly located on the infrastructures from the other PSs, especially in the complex urban scenery by using only SAR images [23,24]; (2) the coverage of a single TSX Stripmap image (30 × 50 km^2^) sometimes cannot cover the entire transportation network (more than 10,000 km) of the typical metropolis in China because it highly depends on the relative positions of the city and the orbit coverage [3,25]. Only a few pioneer works have been conducted to investigate the structural health of infrastructures [2,3,4,5,17,18,19,26,27,28]. However, most of them focused on the analysis of only one to two risk segments at a local scale due to the limited coverage of observation, lacking a systematic comparative analysis of the overall spatial-temporal subsidence evolutions among different types of infrastructures by considering the different operation durations and characteristics. Apparently, instead of the local subsidence of a few segments, the subsidence patterns of the whole infrastructure network are more significant for risk census and government action, which may enable early warnings of damages so as to effectively reduce the potential threats on public safety. Therefore, the accurate health diagnosis especially on different types of infrastructures often proves to be a big challenge, which requires enabling methods to address the above issues.

Aiming at the insufficient of previous studies, the PSI-based subsidence mapping over the entire transportation network of Shanghai metropolis was illustrated in this paper, achieving the city-wide monitoring of the elevated roads, ground highways and underground subways for the first time. The new idea of subsidence comparative analysis among different types of infrastructures was implemented by accurately selecting the PSs along different of types and integrating the different operation durations and structural characteristics into results interpretation. In order to obtain the detailed subsidence distribution of every year, the datasets of each coverage were divided into three one-year-round subsets. Based on the PSs selected combining SAR amplitude and interferometric phase, the precise geolocation and different structural characteristics of infrastructures were introduced to effectively guide more accurate identification and separation of PSs along each type of infrastructures. With the high-density PSs, two neighboring TSX stacks were integrated by joint estimating the measurements in the overlapping area to perform a large-scale subsidence mapping. Instead of simply analyzing the subsidence magnitude and driving factors for the sinking segments as most previous studies usually do, the subsidence maps on each type of infrastructures of each year were generated separately in this study, providing a quickly update for the risk segments inventory map on each type of infrastructures. Moreover, systematical comparative analyzing of subsidence patterns by considering the operation durations and structural characteristics among different types of infrastructures was also implemented, investigating the different subsidence characteristics and possible trigger factors. The experimental results were validated by leveling data and showed a high degree of consistency in terms of subsidence velocities and time-series displacements. Our results revealed that spatial-temporal subsidence patterns on each type of infrastructures are strongly dependent on their operational durations and structural characteristics as well as the variation of the foundation soil layers.

## 2. Study Area and Datasets

### 2.1. Geological Setting

**Figure 1:**
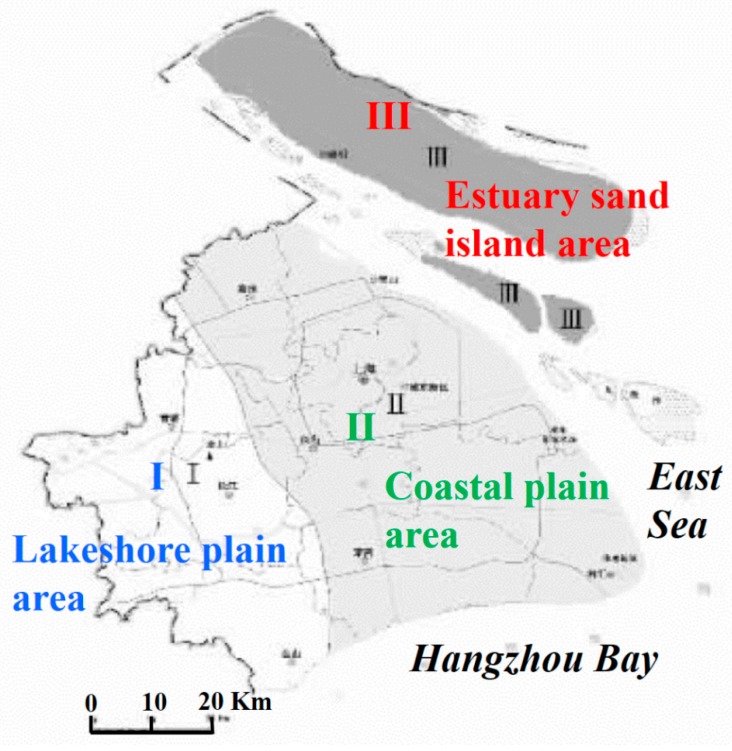
The sketch map of engineering geological division of Shanghai.

Shanghai, as a populated city with a rapid development of infrastructure network, is built on the coastal sand and clay soil that is widely distributed in the upper 40 m below the ground surface [29]. The deposits, in most of the city, are soft sediments that were formed during the Quaternary Era [30]. The silt clay soft soil layer, with the poor engineering geological conditions of high water content, low strength and high compressibility, is frequently used for the underground space development [8]. It is prone to subsidence under the additional loads, which is the main level of land subsidence. The shallow sand and powdery soil layers associated with project construction mainly consist of three parts: shallow layer, middle layer and lower layer, among which the shallow sand layer is generally liquefiable. Therefore, the obvious rheological properties would lead to deformation during the excavation of foundation, affecting the stability of slope [31,32]. According to the changing characteristics of engineering geological conditions, Shanghai can be divided into three engineering geological areas, namely the first engineering geological area (lakeshore plain area), the second engineering geological area (coastal plain area) and the third engineering geological area (estuary sand island area), the sketch map of engineering geological division of Shanghai is shown in Figure 1 as illustrated in [33]. In general, the shallow sand is basically missing in the lakeshore plain area, thus the problem of sand liquefaction is not prominent. However, the natural foundation of the eastern part is worse than the western part which has two hard soil layers. The shallow sand layer and soft soil developed in the coastal plain area, resulting in relative prominent problems of sand liquefaction and soil foundation sinking. The pile foundations in this area vary dramatically due to the cutting of paleo-rivers. The shallow sand layer is extremely abundant in the estuary island area, thus special attention should be paid to the problem of sand liquefaction. Although cut by paleo-rivers, the pile foundation changed slowly due to the relative stable of sand layer in the middle part [33]. 

Based on these soft soil layers, this city has been suffering from severe land subsidence since the last century due to the excessive exploitation of groundwater and rapid urbanization [34,35]. Although the municipal government has taken measures to mitigate this situation, the uneven subsidence of about −15 mm per year (mm/yr) in recent years has still affected and damaged the infrastructures that are undergoing major expansions [36,37]. 

### 2.2. Infrastructures Development

Currently, the total mileage of roads in Shanghai is about 12,300 km. Since 1995, Shanghai has increased the subway network from one single line to 16 subway lines with 336 subway stations in total and 617 km in length. According to the last reports, by the end of 2020 Shanghai plans to extend the subway network to a total number of 22 lines, spanning almost 900 km [3]. Since these infrastructures are located on the deltaic deposit of the Yangtze River, the compaction of the upper soft clay and other anthropogenic factors would lead to considerable deterioration in terms of structural capacity and functionality, endangering the transportation safety. In order to express the deformation characteristics of the large infrastructures more accurately, we divided them into three types based on their elevation: the elevated roads, the ground roads and the underground subways. The investigations of spatial-temporal subsidence variation were then carried out on each type of infrastructure separately before a subsidence comparative analysis among the three types of infrastructures.

### 2.3. InSAR Datasets

**Figure 2:**
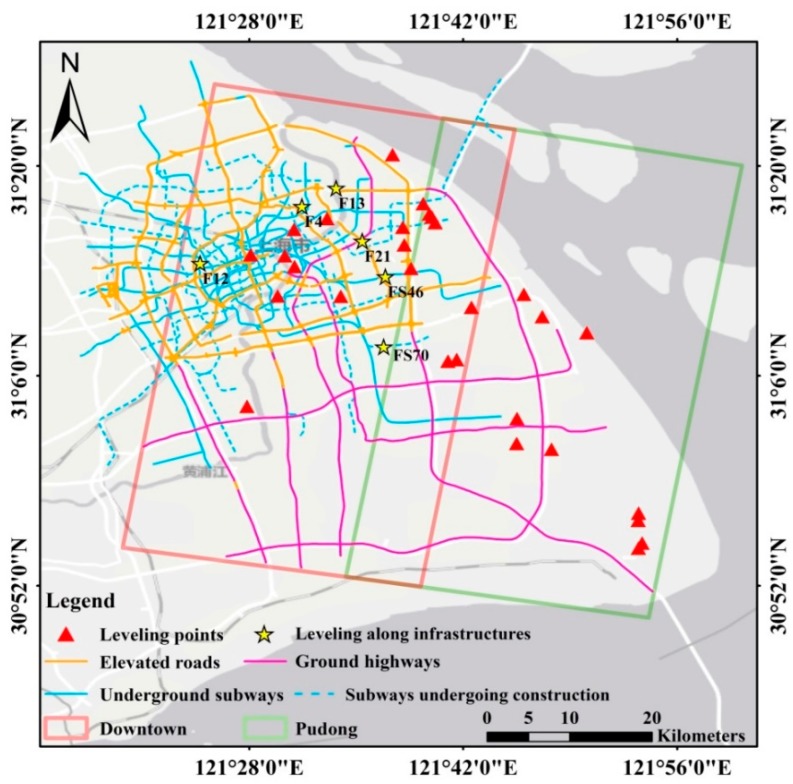
Study area. The red rectangle in the west is the coverage of Downtown area and the green rectangle in the east indicates the Pudong area. The red triangles imply the positions of leveling data and the yellow stars indicate the leveling points along the infrastructures. The orange, purple and blue lines represent the elevated roads, ground highways and underground subways respectively.

To extract the subsidence evolution along these infrastructures, two descending coverages of 70 TSX Stripmap images with an overlapping area of about 300 km^2^ from July 2013 to December 2016 were collected in this study. The spatial resolution of TSX images used in this study is about 3 m and the acquisition time is around 5 am. In order to cover the whole Shanghai city, we tuned the look angle of satellite in the same path to 15 degrees alternate when it flies over Shanghai. Therefore, we obtained two neighboring coverages in Shanghai as portrayed in Figure 2. The red and green rectangles indicate the coverage of the Downtown and Pudong areas respectively. The elevated roads, ground highways and underground subways are marked by the yellow, purple and blue lines respectively. The leveling points expressed by red triangles are used for results validation and the yellow stars along infrastructures are for specific time-series verification. The detailed information of the datasets is shown in Table 1, Table 2 and Table 3. All the datasets showed extremely small perpendicular ($B_{perp}$) and temporal ($B_{temp}$) baselines, indicating high quality results. Specially, the datasets were divided into three one-year-round subsets, with one or two repeated images between two continuous subsets. Therefore, both the annual subsidence patterns on each type of infrastructures and the entire time-series displacements linking by the repeated images can be obtained. The precise geolocation of infrastructures with longitude, latitude and elevation information provided by the Shanghai Institution of Geological Survey was introduced to improve the accuracy of PSs upon the structures. 

## 3. Methodology for Infrastructure Deformation Monitoring

**Figure 3:**
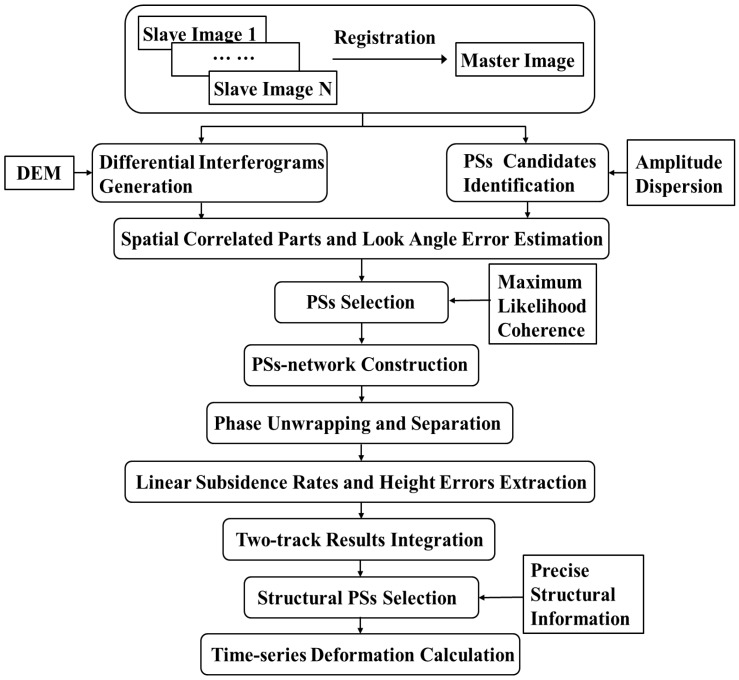
Workflow of the improved PS-InSAR approach.

We briefly review the core idea of traditional PSI technique and specially illustrate our improvements in this work. The whole workflow of the improved PSI approach in our experiment is described in Figure 3. 

Instead of selecting PSs only with the amplitude, phase and coherence as illustrated in most previous research, the precise structural information, including the elevation, geolocation and structural characteristics of infrastructures were integrated into our method to obtain a stack of PSs with optimized accuracy along the structures. Although several literatures have presented various approaches to use the elevation/height data to validate the geolocation of PSs, we aimed at selecting the structural PSs more accurately by using the detailed spatial position analysis between PSs and the specifics structures. It is not only depending on their elevation data but also related to their geolocation information and structural characteristics. The selection strategies of PSs along different types of infrastructures are slightly variant, depending on the detailed spatial position analysis between the PSs and the specific structures. Moreover, the integration of dual-coverage results was implemented by joint estimating the measurements on the transportation infrastructures in the overlapping area after correction with respect to leveling data (shown in Figure 2), allowing a city-wide subsidence mapping on different types of infrastructures in Shanghai.

### 3.1. Structural PSs Selection

From the co-registered SLCs, a set of points with high backscattering intensity throughout the observation period were considered as PS candidates. Based on these candidates, an Amplitude Dispersion Index (ADI) of 0.4 was implemented to keep the PSs with multi-temporal amplitude stability as the traditional PSI technique does [38,39]. However, the ADI based method is only suitable for identifying PSs in areas with dense artificial features, rather than the low coherence areas in Pudong of Shanghai with very few man-made objects and large area of agricultural land [36,37]. Therefore, more information including the coherence and precise structural information should be integrated for the further PSs selection. 

Since the PSs candidates may contain many incoherent points, the filtered spatial correlated components and look angle error were estimated to calculate the maximum likelihood of coherence, which was used to evaluate the phase stability for each PS [26,40]. Then, the ensemble coherence values were used as thresholds to select PSs [40,41]. Finally, two candidate subsets based on amplitude and coherence were combined together to maximize the density of detectable PSs. 

Detailed spatial position analysis of PSs and the specific structures was incorporated to effectively guide more accurate selection and separation of PSs along different types of infrastructures, leading to slightly various selection strategies for specific infrastructures. For the geolocation of infrastructures, in the longitude-latitude plane, PSs falling outside of a local buffer zone along the precise geo-located structure position were excluded in order to screen out the unrelated points. In the elevation direction, the further selection strategies of PSs for infrastructures with various structural characteristics are slightly different. For the elevated roads, the estimated values of PS elevations on the structures were compared to the provided designed height information. The PSs with the elevations exceed three times standard deviation of its surrounding detected points were filtered out because the elevations of points along the structure should be successive. For the ground highways, we kept the PSs exactly on the road surface, which are most probably reflected from the metal railings and street lamps. The PSs selected from the double-bounce signals of surrounding buildings should be removed, because they are from different targets and would obscure the desirable signals. The underground subways however, are invisible in SAR images. Therefore, we cannot detect the real deformation of the PSs exactly on the tunnels and structures but the subsidence of the PSs overlying the geolocation of subways as illustrated in previous studies [3,5,26,36]. Particularly, in this paper, both the points from the land surface and surrounding buildings with low elevations (less than 5 m) were further analyzed because their displacements are most probably resulted from the underground deformation associated with the tunnels and infrastructures.

### 3.2. Two-Coverage Results Integration

**Figure 4:**
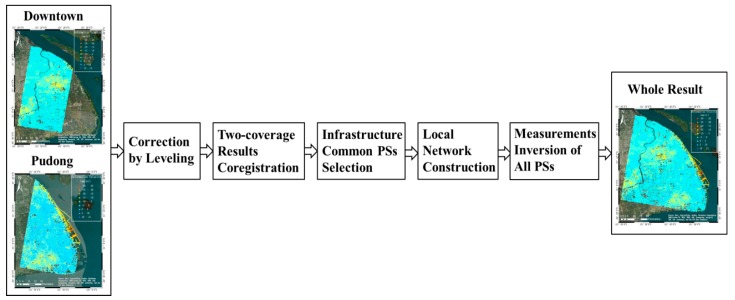
The detail procedure of two-coverages results integration.

Since we aimed to detect the subsidence of infrastructures across the whole Shanghai city, the results separately derived from these two neighboring coverages were further integrated into one subsidence map. The idea of integration has been described in [40], whose essence is to accurately integrate the deformation parameters (velocities, displacements and heights) on common PSs. The procedure of the two-coverage results integration in this paper is illustrated in Figure 4. 

In order to properly combine the deformation products, the measurements were transformed into the WGS84 coordinate system and the results were corrected by the leveling points in the overlapping area. Then, the co-registration of subsidence velocities from Downtown and Pudong areas was implemented by taking the Downtown area as a main track. Due to the different viewing geometries, the PSs in different coverages were not exactly the same. Instead of selecting all the PSs with strong backscattering in both results [42,43,44], only the PS-pairs along the infrastructures with the distance shift less than 0.1 pixels were treated as common PSs candidates so that the subsequent InSAR combination could be applied to homologous targets. Thus, the computational burden was dramatically reduced and the accuracy of measurements along the infrastructures was substantially improved. The PS-pairs with an elevation discrepancy beyond 0.5 m were excluded. Subsequently, the measurements of Pudong area were tuned to those of Downtown area by subtracting the average deviation of the common PSs along the infrastructures. The average deviation and compensated value were calculated by Equations (1) and (2):

(1) $$\Delta v_{pd}=\frac{1}{n}\sum_{i=1}^{n}\left(v_{P_{i}}-v_{D_{i}}\right),$$

(2) $${\tilde{v}}_{P_{j}}=v_{P_{j}}-\Delta v_{\mathrm{pd}}\;(j=1,\;2\ldots \text{the number of Pudong path PSs}),$$

The $\Delta v_{\mathrm{pd}}$ denotes the average deviation between adjacent coverages and *n* indicates the number of common PSs. The $v_{P_{i}}$ and $v_{D_{i}}$ represent the velocity of the *i*th common PS in the Downtown and Pudong area respectively. The ${\tilde{v}}_{P_{j}}$ is the compensated value of the *j*th PS in the Pudong area. After the offset compensation, the final estimates of common PSs ${\bar{v}}_{i}$ in the overlapping area were calculated by Equation (3):

(3) $${\bar{v}}_{i}=\frac{v_{D_{i}}+{\tilde{v}}_{P_{i}}}{2},$$

Considering the errors of estimated parameters are propagated with the distance, we built a local network on the PSs in the overlapping area for velocity inversion, aiming to get continuous and reliable results. For the other PSs in the overlapping area, a Least-Square estimation was applied on the PSs-network to reduce the systematics errors and invert their measurements. Although the time spans of the two coverages are not exactly identical, the average subsidence rates of PSs we integrated were not affected by it because they keep a consistent overall trend over the same period. 

## 4. Results and Validation

### 4.1. Results

**Figure 5:**
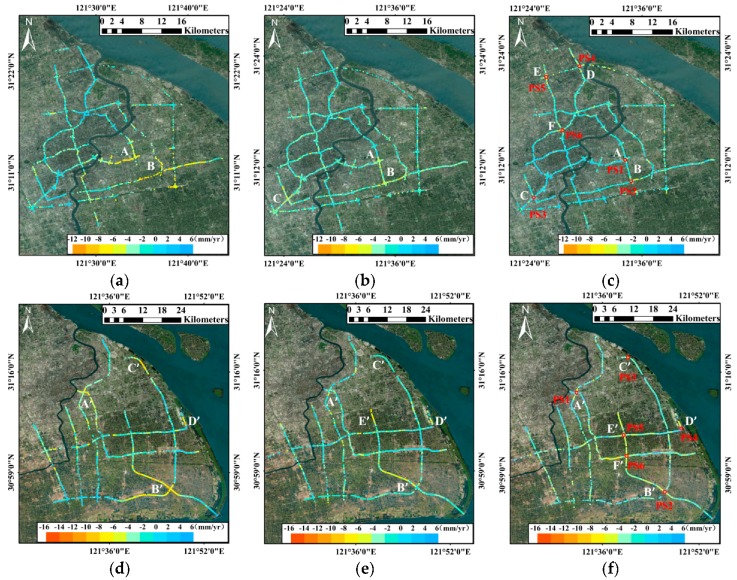

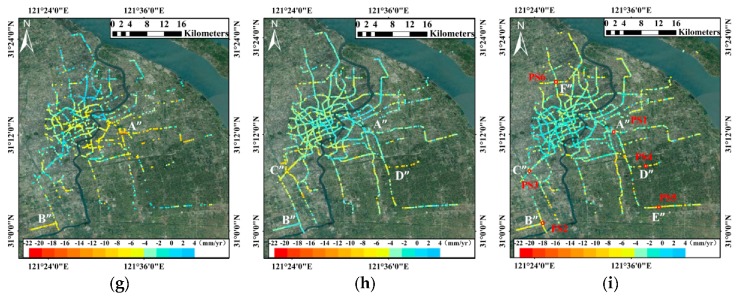
The vertical subsidence velocity maps of three types of infrastructures in Shanghai from 2013 to 2016. (**a**) elevated roads from 2013.8 to 2014.9; (**b**) elevated roads from 2014.9 to 2015.10; (**c**) elevated roads from 2015.10 to 2016.11. (**d**) ground highways from 2013.8 to 2014.9; (**e**) ground highways from 2014.9 to 2015.10; (**f**) ground highways from 2015.10 to 2016.11. (**g**) underground subways from 2013.8 to 2014.9; (**h**) underground subways from 2014.9 to 2015.10; (**i**) underground subways from 2015.10 to 2016.11. Background map: Google Map.

The calculated subsidence velocity maps along the elevated roads, ground highways and underground subways of every year based on the above method are shown in Figure 5, with the subsidence patterns vary spatially and temporally. Interestingly, the entire subsidence on each type of infrastructure progressively decreased from the left (earlier period) to the right (later period) in every row. Although a few new subsidence segments appeared in the suburbs, the major subsidence segments in the main city became stable. According to every column of Figure 5, the overall subsidence levels (indicated by the range of color bars) gradually increased from elevated roads to underground subways during the same observation period. 

### 4.2. Validation

In order to determine whether the subsidence estimates are reliable, the comparison between the measurements of InSAR and leveling was implemented in every year respectively. The validation process consisted of the following two aspects: the widely distributed leveling points for large-scale average subsidence velocity validation and the leveling points located along the infrastructure for specific time-series displacement verification.

**Figure 6:**
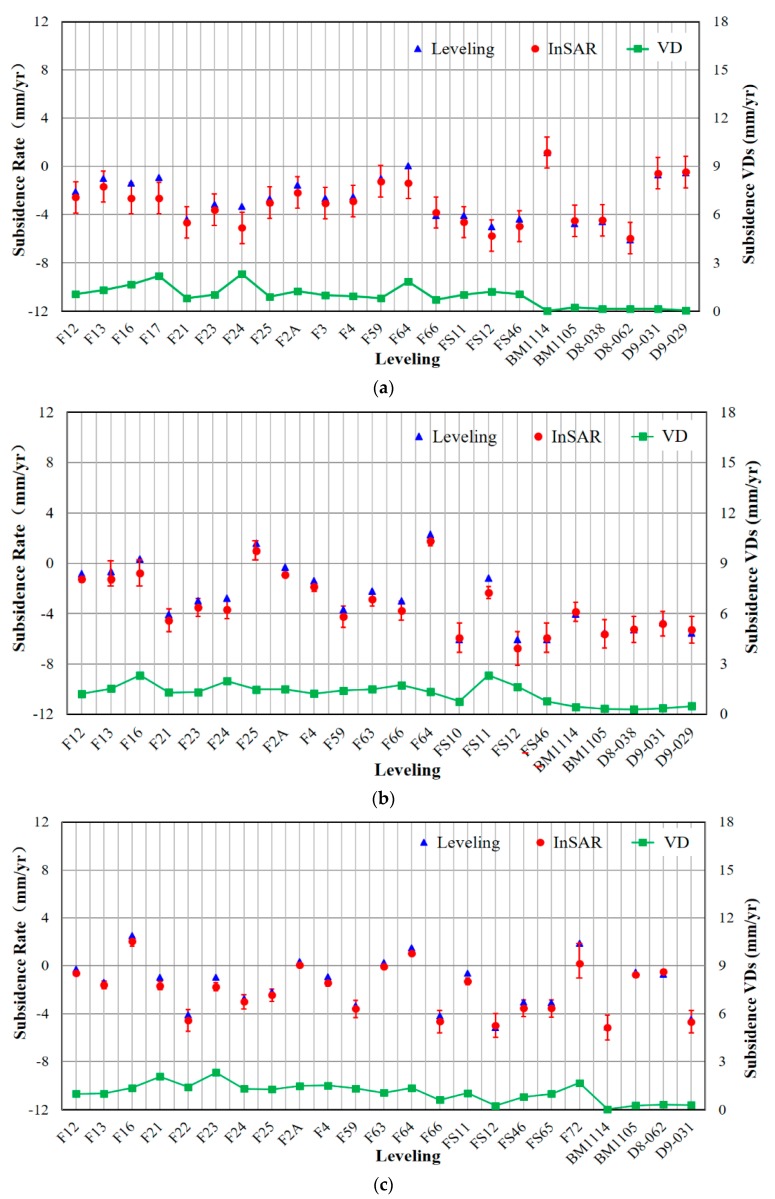
The vertical subsidence velocity comparison between leveling and InSAR in Shanghai from 2013 to 2016. (**a**) 2013.8–2014.9; (**b**) 2014.9–2015.10; (**c**) 2015.10–2016.11. The blue triangles indicate the measurements of leveling and the red circles denote the average rates of PSs within a 50-m buffer zone of the leveling points. The green rectangles represent the subsidence velocity differences (VDs) of the two independent measurements.

We carefully validated the detected subsidence by using 23 leveling points every year. The average vertical subsidence velocities of PSs within a 50-m buffer zone of leveling points (the red circles) were calculated and compared with those of the central leveling points (the blue triangles) in Figure 6. According to the standard for Chinese secondary leveling measurements, the accuracy of leveling data was set to 3 mm/yr. The stability interval of PSs was represented by the red error bars in Figure 6 which are the velocity standard deviation (SDs) of the PSs around the central leveling points. It is worth mentioning that the leveling data named with F and FS are the shallow layered marks without a benchmark on the bedrock, which represent the subsidence between these points and the deepest benchmark. However, the leveling points named BM and D are the surface leveling points that are associated with a bedrock mark, which indicates the total subsidence from the land surface to the bedrock. Since the InSAR technologies detect the surface subsidence, the vertical velocity differences (VDs) of the surface leveling points are smaller than those of shallow layered marks. That is because the shallow layered marks only detect the subsidence of partial deposition layer, whose measurements are smaller than the surface subsidence which include the subsidence of the whole soil layer. Fortunately, the subsidence of the deep solid continental deposition layer is usually quite small. Therefore, the shallow layered marks can also be used to measure the approximate surface subsidence.

The subsidence VDs of the measurements on all the leveling points are less than 3 mm/yr, meaning that no significant difference between these two independent methods. To further compare the subsidence rates obtained by InSAR and leveling, the average errors and SDs of subsidence VDs on all leveling points every year were calculated and described in Table 4. In our experiments, the average errors of subsidence VDs are less than 2 mm/yr and the average SDs of subsidence rates differences are no more than 3 mm/yr, indicating the subsidence estimates obtained by our method are unbiased and reliable. 

**Figure 7:**
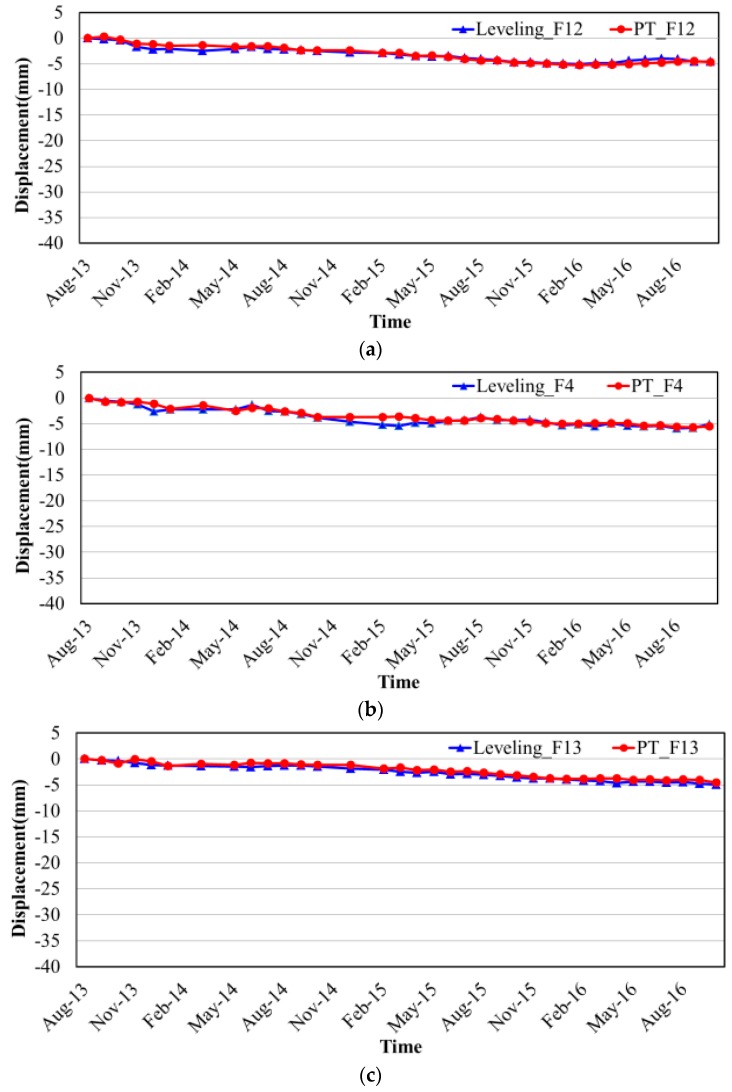

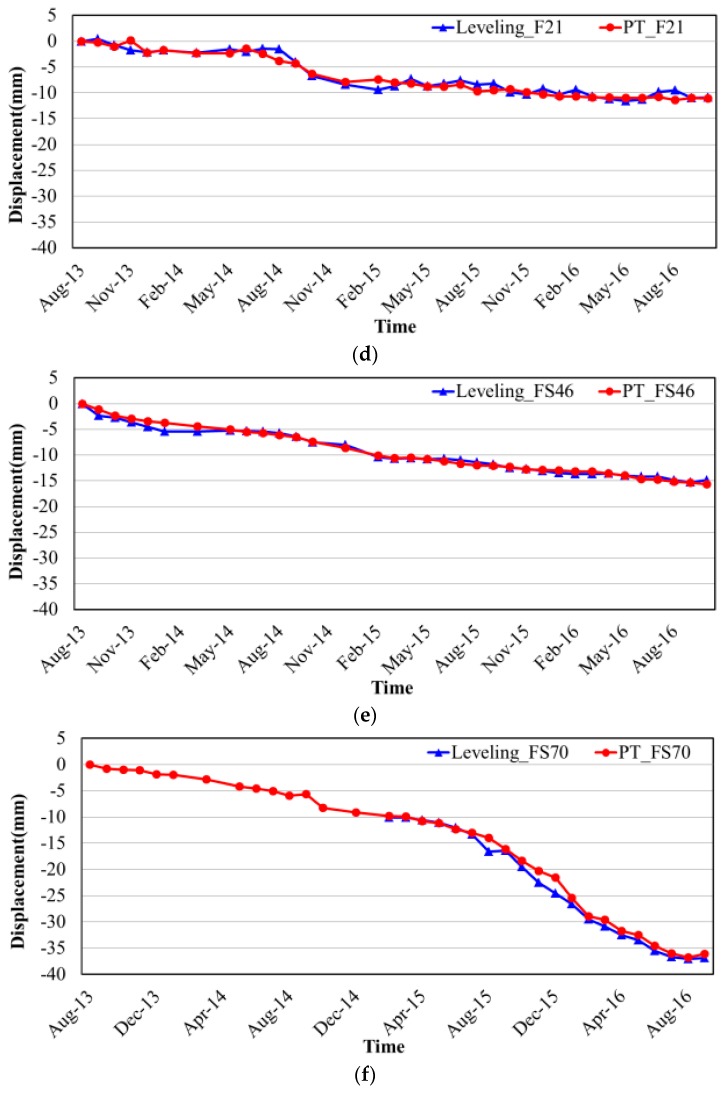
The time-series displacements comparison between PSs and leveling points along infrastructures. (**a**) F12; (**b**) F4; (**c**) F13; (**d**) F21; (**e**) FS46; (**f**) FS70.

In order to further accurately verify the subsidence along these infrastructures, six available leveling points located along the routes were used for the specific time-series comparison as shown in Figure 7. It is obviously that the time-series displacements of the InSAR and leveling results showed a good agreement with each other no matter in the stable segments or the subsidence segments.

## 5. Spatial-Temporal Evolution of Infrastructures Deformation

### 5.1. Elevated Roads

**Figure 8:**
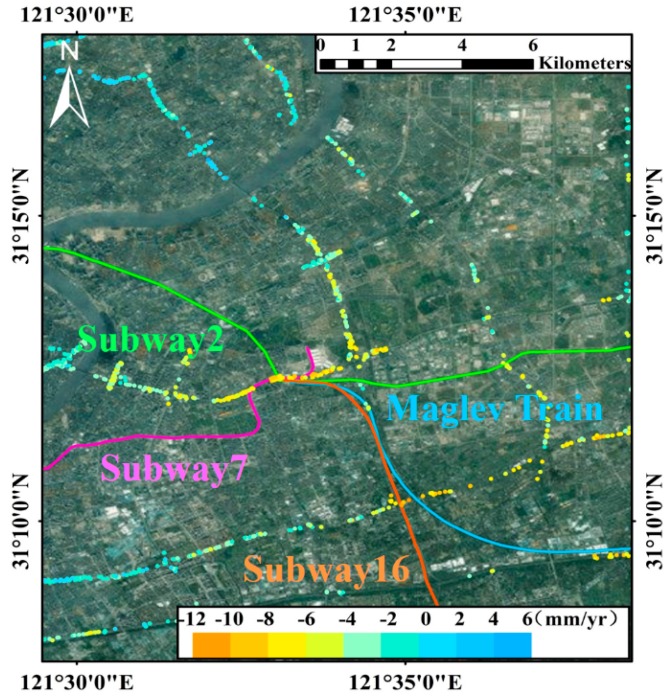
The intersection area among three subways and the maglev train in segment A in the elevated roads. Background map: Google Map.

The elevated roads, mainly distributed in the downtown of Shanghai, are primarily constituted by three ring elevated roads (the Inner Ring, Central Ring and Outer Ring), the North-south Elevated Road, the Yanan Elevated Road and the Yixian Elevated Road. The linear subsidence velocity maps along the elevated roads with the PSs density of around 265 PSs/km^2^ from 2013 to 2016 are shown in Figure 5a–c. The subsidence segments of every year slowly moved from the south towards the north. In Figure 5a, the rapid subsidence, reaching up to −12 mm/yr, occurred in A (the southeast segment of the Inner Ring Elevated Road) and B (the southeast segment of the Central Ring Elevated Road) from August 2013 to September 2014 and a few uplift segments (within 6 mm/yr) appeared in the north and southwest parts of the network. According to our investigation, these two subsidence segments are around the transportation hubs which connect the subway line 7, line 2, line 16 and the Maglev Train as shown in Figure 8. Specially, the subway line 16 was undergoing construction during the observation period. Thus, the subsidence was most probably affected by the construction of underground space, as well as the overlapping traffic loads. In the following year, the overall subsidence along the elevated roads in Figure 5b decreased slightly, indicating the effectiveness of the subsidence controlling measures taken by the government during these years. Both the segments A and B became more stable with the average velocities of −6 and −8 mm/yr respectively. On the contrary, the average subsidence velocity near segment C (the Hongmei Road Overpass) in the southwest rose to about −6 mm/yr, almost catching up with segment A. In Figure 5c, the segments A, B and C in the south experienced downward trends in subsidence rates (less than −4 mm/yr) in the last year. However, three new segments (D, E and F) with increased subsidence rates appeared in the north of the network. Particularly, they are all near the crossroads of two elevated roads which are more likely to suffer from the subsidence due to the overlapping traffic loads.

**Figure 9:**
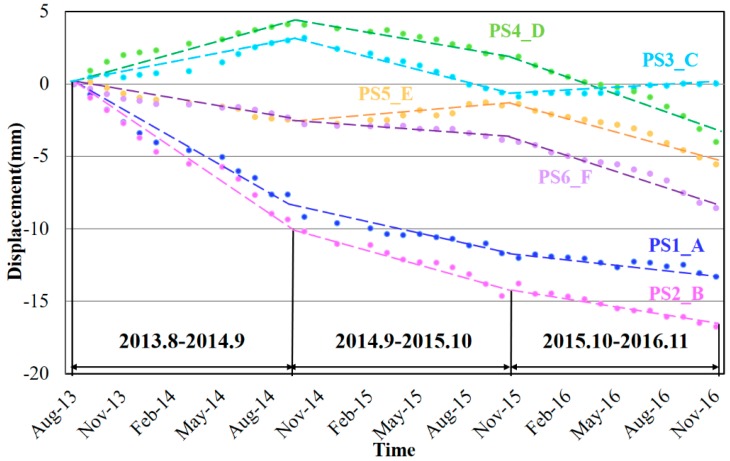
Deformation time-series from 2013 to 2016 of six elevated roads risk segments (A–F) in Shanghai. The dotted lines represent the fitting lines of the time-series displacements.

We calculated the 3-year-round time-series displacements of PSs on segments A–F (marked in Figure 5c) to get more detailed information of the ongoing settlement. For the segment A, B and F, they showed different levels of continuous subsidence during the observation period in Figure 9. Both the subsidence velocities of segment A and B gradually decreased, with a total accumulated subsidence of about 13 and 16 mm respectively. The subsidence rate of segment F slightly increased to about −5 mm/yr but with a much smaller accumulated subsidence of 8 mm. Regarding the segment C, D and E, the total subsidence of them are all less than 6 mm, which are relatively stable with small uplifts and subsidence during the observation periods. Therefore, more attention should be focused on segment A, B and F. Although the subsidence in segment F is still small, the accelerated subsidence velocity is extremely worthy of attention.

### 5.2. Ground Highways

**Figure 10:**
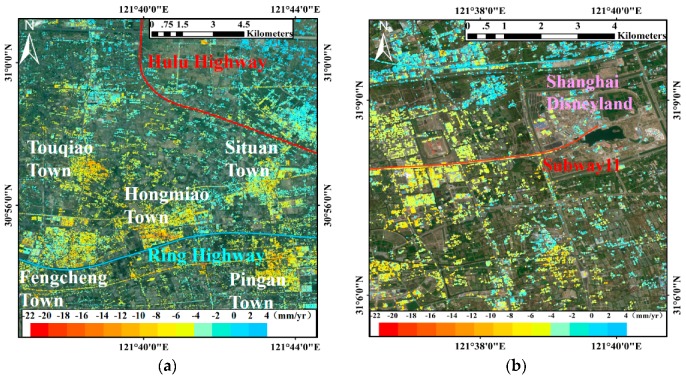
(**a**) The intensive newly developed communities around segment $B^{\prime }$ in the ground highways; (**b**) More PSs selected along segment $D^{\prime \prime }$ (subway 11) in the underground subways after the construction. Background map: Google Map.

The ground highways, mainly distributed in the suburbs of Shanghai, are constituted by the Ring Highway, Hulu Highway, Hujing Highway, Shengjiahu Highway, South Highway and Puxing Highway. Geologically, the research area comprises quaternary deposits with increasing thickness and compressibility from west to east [45]. Therefore, most of the subsidence segments are located in the east part of the network due to the weaker geological foundation in the east. The subsidence velocity maps of ground highways with the average PSs density of about 187 PS/km^2^ are shown in Figure 5d–f. Segments $B^{\prime }$ and $D^{\prime }$ suffered from the most serious subsidence (up to about −14 mm/yr) and they are followed by segments $A^{\prime }$ and $C^{\prime }$ with the slight-to-moderate subsidence rates about −8 mm/yr. Both the segments $A^{\prime }$ and $B^{\prime }$ are the intersections of two roads in the suburbs which surrounded by the newly developed communities (see Figure 10a) without adequate soft soil foundation treatments [46]. Therefore, the superimposed loads from traffic and buildings would lead to subsidence of these segments. Both the segments $C^{\prime }$ and $D^{\prime }$ are located on the ocean-reclaimed soft soil which is unstable and prone to subside. The subsidence velocities of these four segments dropped in the next two years, however, those of segments $E^{\prime }$ and $F^{\prime }$ rose remarkably. 

**Figure 11:**
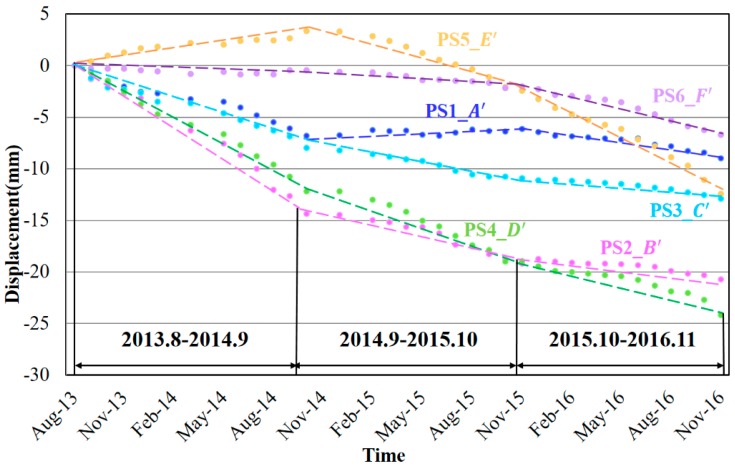
Deformation time-series from 2013 to 2016 of six ground highways risk segments ($A^{\prime }$–$F^{\prime }$) in Shanghai. The dotted lines represent the fitting lines of the time-series displacements.

The time-series displacements of PSs on the six risk segments $A^{\prime }$–$F^{\prime }$ (see Figure 5f) are illustrated in Figure 11 to investigate the long-term subsidence trends. All the six segments subsided during the observation period except for a small uplift of segment $E^{\prime }$ in the first year. Regarding the accumulated subsidence, the segment $D^{\prime }$ near the Pudong Airport ranked the top (about 25 mm) and that was slightly followed by the segment $B^{\prime }$ (approximately 20 mm). Then, both the segments $C^{\prime }$ and $E^{\prime }$ showed the accumulated subsidence around 12 mm and finally, the accumulated subsidence of segment $A^{\prime }$ and $F^{\prime }$ were about 8 mm. Consequently, the segments $D^{\prime }$ and $B^{\prime }$ with the subsidence over 20 mm deserved to be concerned, as well as the segments $E^{\prime }$ and $F^{\prime }$ whose subsidence velocities gradually increased later.

### 5.3. Underground Subways

Except for very few segments above the ground, most of the subway tunnels in Shanghai pass through the extremely soft silty clay underground with poor permeability and high compressibility, increasing the risk to subsidence [47,48]. Although the subway structures and tunnels are invisible in SAR images, it is possible to track the PSs along the path of subway tunnels by looking at the overlying surface subsidence [3,5,26]. That is because the structurally deficient of underground subways would most probably affect the natural support of the overlying strata, resulting in the deformation of the land surface [45]. The subsidence velocities along the routes of underground subways from 2013 to 2016 are demonstrated in Figure 5g–i, indicating a subsidence development trend spatially from the main city to the suburbs. More than a half of the segments suffered from different levels of subsidence in Figure 5g. In the first year, segments $A^{\prime \prime }$ and $B^{\prime \prime }$ stood out as the top two largest subsidence segments with the velocities of −16 and −12 mm/yr respectively. The segment $A^{\prime \prime }$ is the intersection region of maglev train, subway line 7, line 2 and line 16 (undergoing construction). The underground construction and overlapping traffic loads disturbed the geological environments and resulted in the subsidence. The subsidence velocities increased along segments $C^{\prime \prime }$ and $D^{\prime \prime }$ in Figure 4h, however, those of $A^{\prime \prime }$ and $B^{\prime \prime }$ dramatically decreased. Specially, the segment $D^{\prime \prime }$ is the third project of subway line 11 near the new Disneyland in Shanghai. Therefore, it is affected by noise derived from surface scattering changes and no reliable detectable PSs can be extracted when it was under construction (during the first year). After the construction finished, more PSs along this segment (see Figure 10b) were identified with the increasing subsidence which probably due to the post-construction settlement. Afterwards, that subsidence decreased slightly in the last year. Two segments in the newly built subway line 16 ($E^{\prime \prime }$) and subway line 18 ($F^{\prime \prime }$) began to sink from 2015. 

**Figure 12:**
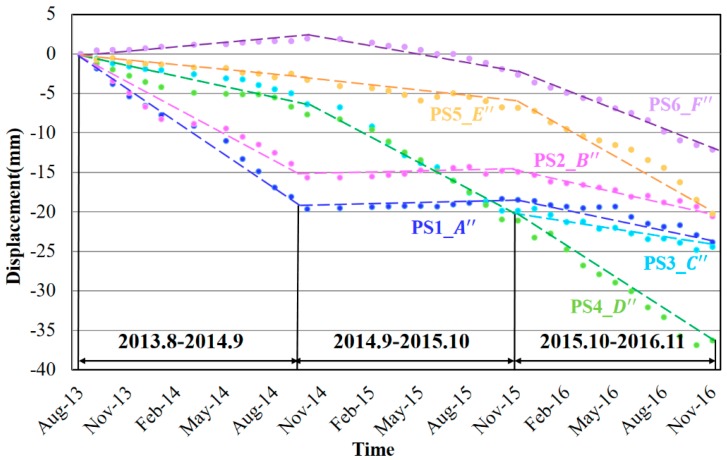
Deformation time-series from 2013 to 2016 of six underground subways risk segments ($A^{\prime \prime }$–$F^{\prime \prime }$) in Shanghai. The dotted lines represent the fitting lines of the time-series displacements.

In order to get a better idea of the subsidence evolutions, the time-series displacements of PSs along segments $A^{\prime \prime }$–$F^{\prime \prime }$ (remarked in Figure 5i) are calculated and illustrated in Figure 12. For segments $A^{\prime \prime }$ and $B^{\prime \prime }$, they remarkably subsided in the first year and then stabilized in the next two years, with the total accumulated settlement of about 18 mm and 15 mm respectively. As the segments $C^{\prime \prime }$ was built later, its deformation behaviors seemed a year later than $A^{\prime \prime }$ and $B^{\prime \prime }$, with two years’ continuous subsidence and then one year’ stability. Regarding the segments $E^{\prime \prime }$ and $F^{\prime \prime }$, they were relatively stable in the first year and began to sink with the rising velocities since the second year. Considering the construction periods and opening dates of the subways, the segments $A^{\prime \prime }$, $B^{\prime \prime }$ and $C^{\prime \prime }$ became steadier after one or two years’ post-construction settlement and soil consolidation. The subsidence velocity of segment $D^{\prime \prime }$ did not decrease due to the post-construction settlement soon after the operation of subway line 11 and the newly built segments $E^{\prime \prime }$ and $F^{\prime \prime }$ are still undergoing the increasing subsidence. Therefore, we can infer that the newly excavated tunnels would firstly affected by the post-construction settlement for one or two years and then gradually became stable.

## 6. Factors for Infrastructure Deformation

### 6.1. Thermal Expansions

It is well known that the X-band interferometric phases are highly influenced by the thermal expansion of concrete and steel structures resulting from the ambient temperature disparities [4,49,50,51]. In this study, dense PSs were successfully found on some significant transportation hubs and millimetric seasonal displacements were detected on these PSs. In order to interpret the observed summer-winter seasonal cycles, we analyzed the SAR measurements together with ancillary data such as the temperature measurements. 

**Figure 13:**
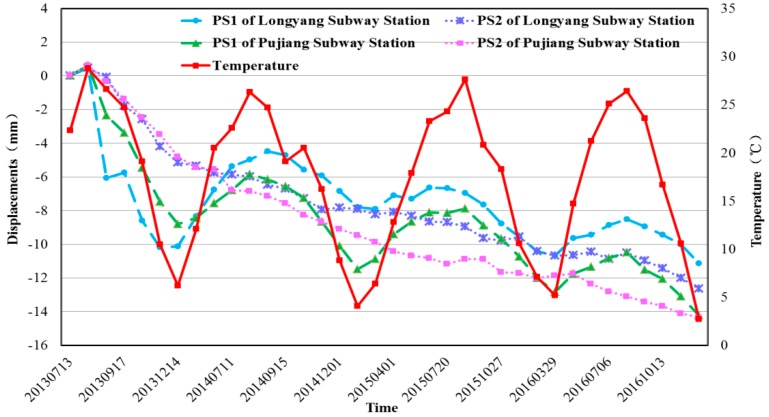
Time-series displacements of Subway Stations: the blue and purple lines indicate the time-series displacements of PS1 and PS2 in Longyang Subway Station and the green and red lines represent the time-series displacements of PS1 and PS2 in Pujiang Subway Station. The red line denotes the corresponding temperature records.

**Figure 14:**
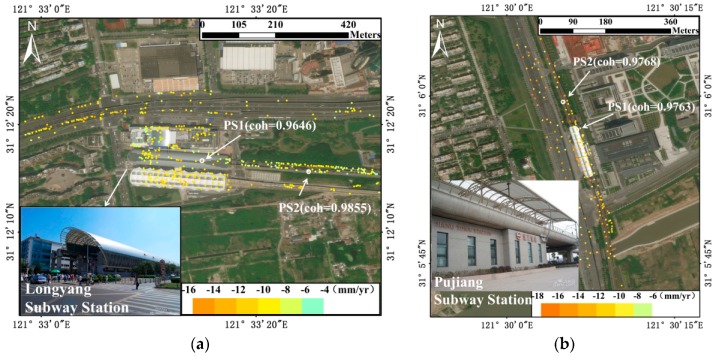
The positions of PSs in Figure 13. (**a**) PSs in Longyang Subway Station; (**b**) PSs in Pujiang Subway Station. Background map: Google Map.

Taking Longyang and Pujiang Subway Stations as example, Figure 13 shows the time-series displacements of high coherence PSs (shown in Figure 14a,b) on the two subway stations. Both the general movements of PSs located on the top of the subway station buildings (PS1s in Figure 14) showed strong correlation with temperature. When the temperature is high in the summer days, the expansion of the buildings results in the movements of slower subsidence or even slight uplift. By contrast, the lower temperature leads to contraction of the buildings, accelerating the subsidence movements during the winter. The PSs located on the road (PS2s in Figure 14), on the other hand, experienced linear subsidence movements during the observation period, showing ignorable general movements due to thermal expansions.

**Figure 15:**
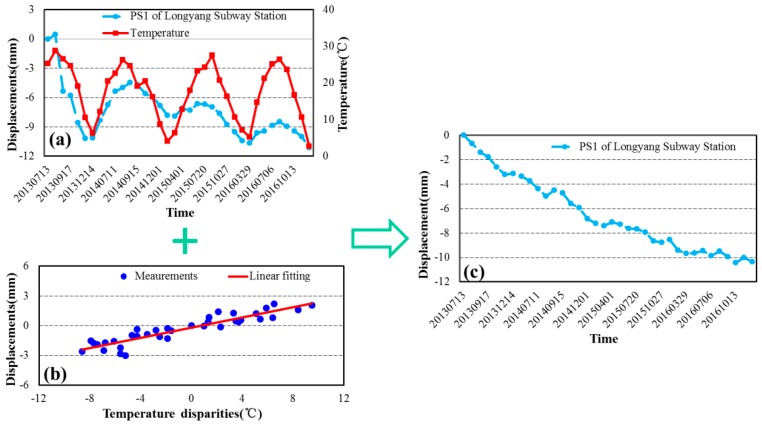
Time-series displacements of PS1 at Longyang Subway Station. (**a**) total observation displacements and temperature records; (**b**) thermal expansions linear fitting results; (**c**) deformation of interest after removing thermal expansions.

**Figure 16:**
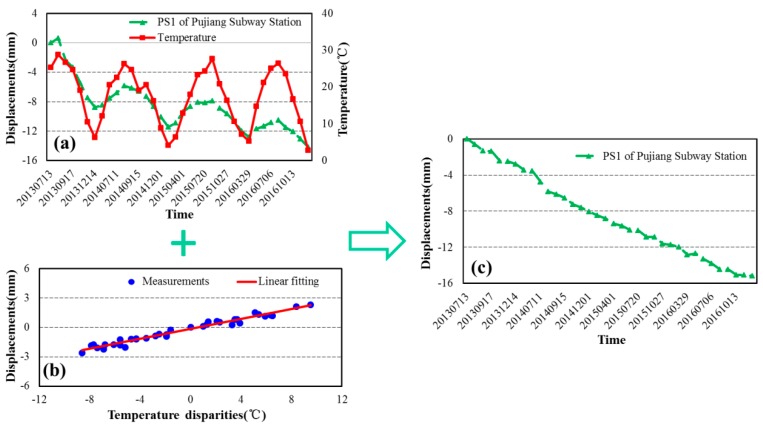
Time-series displacements of PS1 at Pujiang Subway Station. (**a**) total observation displacements and temperature records; (**b**) thermal expansions linear fitting results; (**c**) deformation of interest after removing thermal expansions.

When we use the time-series displacement of a given PS for deformation analysis, the residual phase term still remained and was considered as an estimation of the seasonal displacements contribution [18,28]. Based on the above analysis, a linear regression model was used to quantitatively investigated thermal expansion of transportation hubs. The modelling results and the final irreversible deformation of the two PSs located on the top of Longyang and Pujiang Subway Stations are illustrated in Figure 15 and Figure 16 respectively. 

According to Figure 15 and Figure 16, both the time-series displacements of these two PSs experienced good linear fitting results with the temperature disparities. After removing the thermal expansions, the deformation of interest of both PSs showed linear displacements during the observation period. Therefore, we can infer that the thermal expansions due to the temperature disparities is one of the factors that caused the deformation especially for the transportation hubs.

### 6.2. Geological Features

The widespread soft soil in Shanghai can be divided into nine engineering geological layers (①–⑨), described in Table 5, based on their origins, geological ages, soil behaviors and physical and mechanical properties [30,31]. Constructing infrastructures on such soft clay soil, the poor engineering geological characteristics, such as uneven local consolidation and overdue elastic deformation, would probably destroy the structures and lead to frequently maintenance [3,12,38]. According to our results, the different components and lithology of the underlying soil are important factors influencing the subsidence of the infrastructures. 

According to the distributed range of the engineering geological layers in Table 5, most of the soft soil (①–⑤) are poor holding layers for foundation and are distributed in the estuary island and the eastern coastal belt. The top soil of the eastern coastal area is mainly composed of loose and newly deposited soil layer. Lack of the hard soil layers as good natural foundation, it is not suitable to be directly used as the foundation holding layer [52]. Therefore, most of the subsidence occurred in the Pudong District in the east. A number of buried crisscrossed ditches and paleo-rivers near the Pudong Airport also lead to a worse engineering geological condition and a great change in the stratigraphic [38], increasing the uneven subsidence and slope instability on the roads and tunnels. Therefore, some coastal sections, such as segment $D^{\prime }$, $B^{\prime }$ and $D^{\prime \prime }$ experienced large cumulative subsidence. 

For the soil lithology, since the main shallow layer of Shanghai is the saturated liquefied sandy layers, such as layers ①–⑤, with the potential risk of water leakage, the subway tunnels crossing these layers showed large cumulative subsidence. However, for the elevated roads with the deep pile foundations, the lower strata (the layers ⑦–⑨) are good holding layers for deep foundations and are used for supporting layers [30,31]. Consequently, most of the elevated roads are more stable than the ground and underground infrastructures based on shallow foundations.

### 6.3. Regional Land Subsidence and Construction

As the linear features usually cross the surface and underground soft soil, the change of urban geological environment would inevitably affect the subsidence along the infrastructures. For example, it is obviously that the subsidence of segment $B^{\prime }$ is basically consistent with the subsidence of the surrounding towns as shown in Figure 12a. Moreover, the comparison between widely spread leveling points and PSs also showed a good agreement in Figure 6, indicating the subsidence along the infrastructures affected by regional land subsidence.

Recently, the construction has been a new subsidence factor that cannot be ignored. For example, the segment A is an intersection area of three subways and the maglev train as shown in Figure 10. Since the subway line 16 was under construction from 2013 to 2014, an obvious subsidence can be observed in segment A in the first year, followed by a stable trend in the next two years. The segment $D^{\prime \prime }$ of subway 11 near the Shanghai Disneyland was under construction during the observation period, it subsided during the construction period and after operation. That is because the excavation and unloading of the soil layer during the construction released the outside soil stress, leading to the displacement of tunnels and surface [53]. The long-term underground space excavation, especially the corresponding engineering drainage measures, removed the natural support from the overlying strata and changed the initial equilibrium state, resulting in the surface subsidence [43]. Therefore, the infrastructures surrounded by the construction projects are high-risk sections.

## 7. Comparison and Discussion

In order to compare the subsidence status of the elevated roads, ground highways and underground subways, the subsidence velocity range and subsidence PSs ratio along these structures are described in Table 6. The whole range of subsidence rate on the elevated roads gradually decreased from [−12,6] to [−10,5] mm/yr. Meanwhile, the percentage of subsidence PSs began at 54.46% and quickly reduced to 41.31% by 2015 before slightly dropped to 36.54% in the end. As for the ground highways, the subsidence velocity range also deceased a little from [−16,6] to [−14,6] mm/yr and the subsidence PSs ratio fell rapidly from 58.2% in 2013 to 42.5% in 2016. Similarly, those of underground subways both declined from [−16,4] to [−14,4] and from 60.29% to 45.92% respectively. It is clearly that both the range and ratio of subsidence on these infrastructures decreased gradually, indicating an upward trend in the stability. Clearly, the elevated roads suffered from the slightest subsidence followed by the ground highways, while the subsidence along the underground subways is the most serious. The possible reasons for this phenomenon can be summarized as follows:

(1). The construction and opening dates of infrastructures. As most of the elevated roads in Shanghai were built before 2002, they are stable after the long-term post-construction settlement and effective management and maintenance. Only the segment A and B, with the most severe subsidence, were built later than 2010, which may still undergo the gentle soil consolidation after construction. The ground highways, built around 2008, suffered from a more severe subsidence than the elevated roads during the observation period. Since most of the underground subways were built later than 2007 and some of them are still undergoing construction, they are seriously affected by the soft soil consolidation caused by the frequent tunnel excavation and traffic loads. Therefore, the subsidence of the large infrastructures is strongly related to their construction and opening dates: the earlier built segments are usually more stable than the later constructed segments. 

(2). The structure type of infrastructures. It is widely believed that the elevated roads are supported by deep pile foundations with the mature design specifications, which can not only sustain a larger load on the upper structure but also slow down the subsidence relatively. Thus, the displacement along the elevated roads is the smallest. The ground highways, located on the vulnerable compressible alluvial plain, are probably to subside due to the static loads from transport equipment and dynamic loads from moving traffic. Lack of the stable support from the deep pile foundation, their settlements are larger than the elevated roads. The ground highways’ settlements however, are still smaller than those along the underground subways buried into the shallow soft clay. The underground soft layer subgrade, without good holding layers, is prone to subsidence due to the long-term traffic loads. Moreover, the additional stressors from the overlying strata, such as the soil self-weight, superimposed loads from ground equipment, may also accelerate the subsidence along the underground subways. Consequently, the subsidence along the large infrastructures are closely related to their structure types: the elevated segments with deep pile foundations are more stable than the ground segments based on shallow foundations, while the underground segments buried into the soft soil suffered from the most serious subsidence. 

(3). The variation of the foundation soil layers. Combining the geological setting (see Figure 1) and the distribution of infrastructures (see Figure 2) in Shanghai, our results are related to the variation of the foundation soil layers. Considering the elevated roads are mainly built on the lakeshore plain area and coastal plain area with the relatively thin silty clay, only a slight subsidence (with a maximum rate of 12 mm/yr) was visible on a few segments. However, the ground roads and underground subways are mainly distributed in the coastal area and some of them extended into the estuary island area, which are mainly consist of silty clay and sandy silt. Their foundations are weaker than those of elevated roads due to a reduced consolidation time as well as the softer geology formed by blown sand and backfill, resulting in more severe subsidence especially for those segments that are most proximal to the coast, with maximum rates of 16 mm/yr and 22 mm/yr respectively. 

## 8. Conclusions and Outlook

In this study, we investigated the potential of an improved PSI analyses approach for long-term subsidence monitoring of large infrastructures, demonstrating how SAR data can provide useful information for their management and maintenance. Our study shows that the precise structural information can guide a more accurate structural PSs selection by a spatial position analysis, facilitating the calculation of reliable results. After correcting by the leveling data in the overlapping area, the two-coverage results were integrated by joint estimating the measurements on the overlapping area, achieving a city-wide subsidence monitoring of Shanghai. Compared by the leveling data, the SDs less than 3 mm/yr indicated that our results are reliable. The comparison and discussion of the accumulated displacements and spatial-temporal patterns of subsidence along different types of infrastructures were carried out to investigate the long-term subsidence trends and possible trigger factors. 

The spatial distribution and magnitude of subsidence gradually developed from downtown to suburbs, which is probably because the population and associated human activities in Shanghai continues to grow and moves from the city to the suburbs. The subsidence on different types of infrastructures indicated that the subsidence distribution is strongly related to their structural types, expressing as the average subsidence of the ground highways smaller than those of underground subways located in shallow soft soil and in turn larger than those of the elevated roads with deep pile foundations. Additionally, some segments near the coastal area suffered from severe subsidence due to a short consolidation time of their foundations.

In terms of the time-series displacements analysis, the earlier construction sections with long-term settlements and effective management are usually more stable than the later built sections. Although the present-day accumulated settlements of some segments are not severe, there are increasing trends in their subsidence rates. Therefore, the monitoring is still warranted on these segments to avoid the further progress of subsidence. According to the statistics of the subsidence range and subsidence PSs ratio for the three years, the decreasing levels of subsidence are observed in all the three types of infrastructures.

Such observations of engineering-scale subsidence can help engineers detecting the existing subsidence and evaluating the current status on the major infrastructures in a fast and reliable way. Interpretation of subsidence and analysis of its mechanism carried out in this study indicating the broad application prospects of PS-InSAR on the urban ground and underground infrastructures subsidence monitoring and management.

## Figures and Tables

**Table 1:** Basic information for TSX Stripmap images in Shanghai from 2013 to 2014: acquisition date (Date), perpendicular ($B_{perp}$) and temporal ($B_{temp}$) baselines. Master image of Downtown and Pudong are 20140312 and 20140323.

Downtown				Pudong			
No	Date	$B_{perp}$ (meters)	$B_{temp}$ (days)	No	Date	$B_{perp}$ (meters)	$B_{temp}$ (days)
1	20130713	78.7	−242	1	20130815	34.3	−220
2	20130804	31.6	−220	2	20130928	−194.5	−176
3	20130826	9.7	−198	3	20131111	70.7	−132
4	20130917	−236	−176	4	20131203	16.5	−110
5	20131009	−78.6	−154	5	20131225	47.5	−88
6	20131122	−87.4	−110	6	20140105	217.2	−77
7	20131214	74.1	−88	7	20140323	0	0
8	20140312	0	0	8	20140528	106.4	66
9	20140517	69.2	66	9	20140630	190.1	99
10	20140711	58.7	121	10	20140722	117.4	121
11	20140802	125.8	143	11	20140813	36.5	143
12	20140824	149.8	165	12	20140904	18.5	165
13	20140915	−116	187	13	20140926	−52	187

**Table 2:** Basic information for TSX Stripmap images in Shanghai from 2014 to 2015: Date, $B_{perp}$ and $B_{temp}$. Master image of Downtown and Pudong are 20150310 and 20150216.

Downtown				Pudong			
No	Date	$B_{perp}$ (meters)	$B_{temp}$ (days)	No	Date	$B_{perp}$ (meters)	$B_{temp}$ (days)
1	20140915	134.2	−176	1	20140813	98.3	−176
2	20141007	−132.2	−154	2	20140904	21.3	−154
3	20141029	−38.8	−132	3	20140926	3.6	−132
4	20141201	86.8	−99	4	20141018	−72.5	−121
5	20141223	43.6	−77	5	20141109	105.4	−99
6	20150310	0	0	6	20141212	158.3	−66
7	20150401	301.2	22	7	20150216	0	0
8	20150515	−6.1	66	8	20150504	66.1	66
9	20150617	33.1	99	9	20150606	−4.1	99
10	20150720	−25.3	132	10	20160709	−47	132
11	20150822	−96.6	165	11	20150811	24.7	165
12	20150924	−83.8	198	12	20151016	−11.1	231
13	20151027	72.6	231	13	20151118	89.3	264

**Table 3:** Basic information for TSX Stripmap images in Shanghai from 2015 to 2016: Date, $B_{perp}$ and $B_{temp}$. Master image of Downtown and Pudong are 20160501 and 20160512.

Downtown				Pudong			
No	Date	$B_{perp}$ (meters)	$B_{temp}$ (days)	No	Date	$B_{perp}$ (meters)	$B_{temp}$ (days)
1	20150924	−78.1	−220	1	20151016	−151.5	−209
2	20151027	−65.5	−187	2	20151118	−185.5	−176
3	20151129	25.5	−154	3	20151210	−82	−154
4	20151221	13	−132	4	20160101	−119.9	−132
5	20160329	110.2	−33	5	20160203	−212.6	−99
6	20160501	0	0	6	20160409	−56.9	−33
7	20160603	78.6	33	7	20160512	0	0
8	20160706	208.3	66	8	20160614	9.1	33
9	20160808	171.3	99	9	20160717	−132.5	66
10	20160910	47.4	132	10	20160819	14.1	99
11	20161013	73.6	165	11	20160921	−103	132
12	20161115	−90.1	198	12	20161024	−100	165
13	20161218	−112.2	231	13	20161126	66.5	198

**Table 4:** Comparison of InSAR and Leveling results from 2013 to 2016.

Time	Average Error of Subsidence VDs (mm/yr)	Average SDs of Subsidence VDs (mm/yr)
2013.8–2014.9	1.74	2.55
2014.9–2015.10	1.53	2.46
2015.10–2016.11	1.39	2.30

**Table 5:** The division of engineering geologic layers in Shanghai.

Depth (m)	Layer Number and Lithology	Distributed Range	Foundation Conditions
0–5	①Plain soil; ②_1_, ②_2_ Clay layer	Whole area	Poor shallow holding layer
−5–0	②_3_ Sandy, silt layer	Estuarine island, eastern coast belt, alluvial plain	Prone to quicksand
−8–3	③Silty clay layer	Widely distributed except the estuary island	Poor engineering geological characteristics
−20–3	④Mucky clay layer	Widely distributed	Poor holding layer for foundation
−45–−12	⑤Clay layer	Widely distributed especially the paleo-rivers	Poor holding layer for foundation
−25–−20	⑥First hard soil layer	Widely distributed except the paleo-rivers	Inappropriate for foundation
−40–−15	⑦The second Sandy layer	Widely distributed except the estuary island and paleo-rivers	Good holding layer for building
−60–−35	⑧Clay layer	Lakes and plains	Good holding layer for building
−100–−75	⑨The third sandy layer	Widely distributed	Good holding layer for skyscrapers

**Table 6:** Comparison of subsidence on different types of infrastructures from 2013 to 2016: the whole range subsidence velocity (Range) and the ratio of subsidence PSs (Ratio).

Time	Elevated Roads		Ground Highways		Underground Subways	
	Range (mm/yr)	Ratio (%)	Range (mm/yr)	Ratio (%)	Range (mm/yr)	Ratio (%)
2013.8–2014.9	−12~6	54.46	−16~6	58.2	−16~4	60.29
2014.9–2015.10	−12~5	41.31	−16~5	47.1	−16~4	50.57
2015.10–2016.11	−10~5	36.54	−14~6	42.5	−14~4	45.92
